# Human factors/ergonomics to support the design and testing of rapidly manufactured ventilators in the UK during the COVID-19 pandemic

**DOI:** 10.1093/intqhc/mzaa089

**Published:** 2020-08-11

**Authors:** Sue Hignett, Janette Edmonds, Tracey Herlihey, Laura Pickup, Richard Bye, Emma Crumpton, Mark Sujan, Fran Ives, Daniel P Jenkins, Miranda Newbery, David Embrey, Paul Bowie, Chris Ramsden, Noorzaman Rashid, Alastair Williamson, Anne-Marie Bougeard, Peter MacNaughton

**Affiliations:** School of Design & Creative Arts, Loughborough University, Loughborough, LE11 3TU, UK; The Keil Centre Ltd., Edinburgh, EH3 8HQ, UK; Healthcare Safety Investigation Branch, Farnborough, GU14 0LX, UK; Healthcare Safety Investigation Branch, Farnborough, GU14 0LX, UK; Network Rail, London, NW1 2DN, UK; Systems-Concepts Ltd., London, WC1X 8DP, UK; Human Factors Everywhere Ltd, Woking, GU21 2TJ, UK; West Midlands Academic Health Science Network, Birmingham, B15 2TH, UK; DCA Design International, Warwick, CV34 4AB, UK; Inspired Usability Ltd., Knaresborough, HG5 8HT, UK; Human Reliability Associates, Wigan, WN8 7RP, UK; NHS Education for Scotland, Glasgow, G3 8BW, UK; The Chartered Society of Designers, London, SE1 3GA, UK; Chartered Institute of Ergonomics & Human Factors, Stratford-upon-Avon, B95 6HJ, UK; University Hospitals Birmingham NHS Foundation Trust, Birmingham, B15 2TH, UK; University Hospitals Plymouth NHS Trust, Plymouth, PL6 8DH, UK; Faculty of Intensive Care Medicine, London, WC1R 4SG, UK

**Keywords:** ergonomics, mechanical ventilators, standards, safety, design, usability

## Abstract

**Background:**

This paper describes a rapid response project from the Chartered Institute of Ergonomics & Human Factors (CIEHF) to support the design, development, usability testing and operation of new ventilators as part of the UK response during the COVID-19 pandemic.

**Method:**

A five-step approach was taken to (1) assess the COVID-19 situation and decide to formulate a response; (2) mobilise and coordinate Human Factors/Ergonomics (HFE) specialists; (3) ideate, with HFE specialists collaborating to identify, analyse the issues and opportunities, and develop strategies, plans and processes; (4) generate outputs and solutions; and (5) respond to the COVID-19 situation via targeted support and guidance.

**Results:**

The response for the rapidly manufactured ventilator systems (RMVS) has been used to influence both strategy and practice to address concerns about changing safety standards and the detailed design procedure with RMVS manufacturers.

**Conclusion:**

The documents are part of a wider collection of HFE advice which is available on the CIEHF COVID-19 website (https://covid19.ergonomics.org.uk/).

## Introduction

The COVID-19 pandemic has led to a massive demand for Intensive Care Unit (ICU) facilities, with healthcare providers working to increase the surge capacity of hospitals. To respond to the anticipated demand, the UK Government called for UK manufacturers to increase the number of available ventilators through a process of rapid manufacturing [1]. There were specific challenges, including manufacturers with little experience of healthcare or ventilators, a trade-off between regulatory control, international standards, rapid manufacturing and design for users with less experience of using ventilators.

In the UK, National Health Service design has been accepted as an important component in patient safety since the 2000s [2]. Internationally, a Usability and Human Factors/Ergonomics (HFE) standard for medical device development was established in 2007 [3] and was adopted in the UK in 2017 to address the ‘errors in use leading to patient harm … Such errors may be due to poor device design, particularly where a complex user interface is involved. Medical devices, such as infusion pumps, ventilators, … are recognised as potentially having use-related design issues that can result in problems’ [4]. To support the call for Rapidly Manufactured Ventilator Systems (RMVS; [5]), the Chartered Institute of Ergonomics & Human Factors (CIEHF) produced guidance to help and support manufacturers through the requirement for formative usability testing. It was ‘accepted that full demonstration of compliance to ISO 80601-2-12:2020 is unrealistic in the time frame required for development’ and that when ‘the current emergency has passed these devices will NOT be usable for routine care unless they have been CE marked through the Medical Device Regulations’ [5].

**Figure 1:**
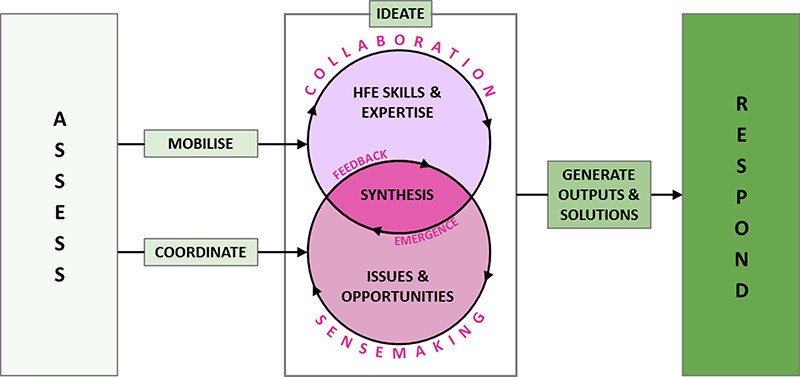
CIEHF response: assess, mobilise and co-ordinate, ideate, generate outputs and solutions, respond.

This paper describes the response process by the CIEHF to develop rapid advisory guidance documents, which was circulated by the UK Government to all RMVS manufacturers. Figure 1 provides a representation of how the CIEHF responded to COVID-10 for the design of ventilators and other projects (https://covid19.ergonomics.org.uk/).

1. **ASSESS**: assess the COVID-19 situation and decide to formulate a response.
2. **MOBILISE AND COORDINATE**: mobilise and coordinate HFE specialists.
3. **IDEATE**: HFE specialists collaborate to identify, analyse issues and opportunities and develop strategies, plans and processes.
4. **GENERATE OUTPUTS AND SOLUTIONS**: outputs and solutions were produced.
5. **RESPOND**: response includes targeted support and guidance.

## Principles of HFE in ventilator design and operation

**Figure 2:**
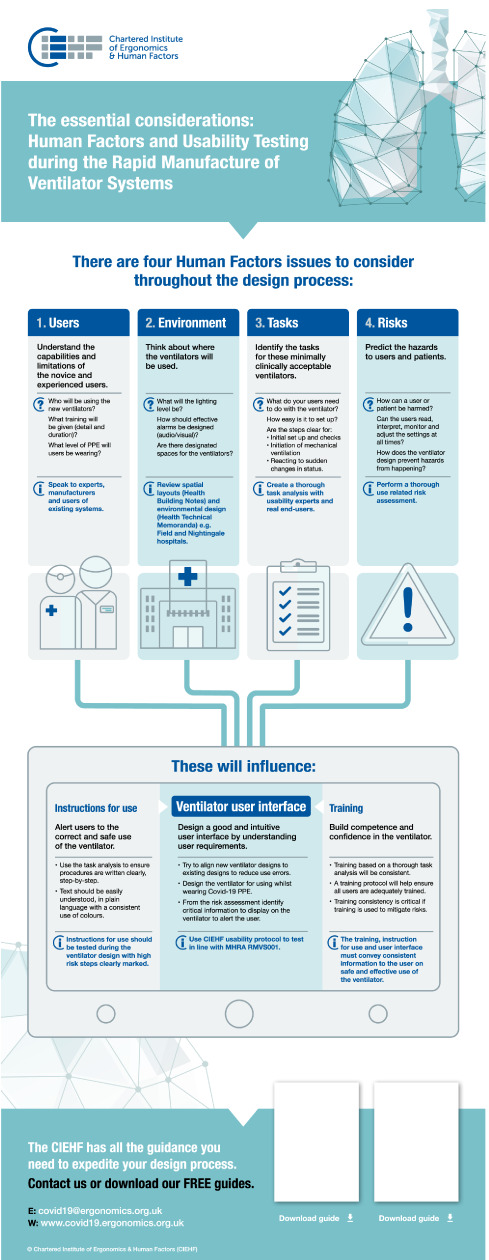
CIEHF guidance infographic.

The first rapid project provided guidance on basic HFE principles (Figure 2). The aim was to support RMVS manufacturers with a structured, yet simple, process for the design of the user interface and instructions for use, and with the development of training based on consideration of users and use environment, the tasks and the associated risks [6]. Each principle was explained clearly using plain language and key learning points. This was followed with a more detailed protocol for usability testing, including patient profiles and clinical test scenarios [7].

## User interface

It was recommended that, where possible, the new ventilator designs should be aligned to existing designs to support existing operational mental models, allow rapid learning and reduce use errors.

The user interface should be intuitive with buttons/controls spaced to minimise accidental operation. There should be informative feedback to users, which is informed by a risk analysis to identify any required warnings or alarms for critical steps and/or unsafe situations. Alarm design should consider the environment(s) of use and be audible in a noisy critical care environment, the potential for alarm fatigue due to multiple alarm systems, as well as lighting at different times of day/night [8]. Generally, if a situation does not require a user action, an alarm should not be used but should instead just display information indicator (feedback). Generic heuristics for interface design quality included consistency of the layout (e.g. colour-coding), transparency about device status and reducing the number of items a user needs to remember.

The physical design recommendations included ensuring that physical connectors were easily recognisable and worked across settings. To design for relocating the ventilator, the weight should be considered, with easy repositioning/adjustments to avoid musculoskeletal health risks to staff (including the adjustment of screens and displays). Retractable cables could reduce trip hazards in the bed space and for storage.

To reflect the different use during the COVID-19 pandemic, it was recommended that manufacturers design interfaces for users wearing personal protective equipment (PPE). This includes eye cover (safety glasses, safety goggles), face cover (surgical mask, face visor), body cover including surgical gowns (with and without sleeves), plastic aprons, one-piece disposable protection suit (and possibly a full gas-tight protection suit) and hand cover with two layers of gloves sticky taped onto the sleeves of gowns in between the layers.

## Tasks

A range of operational tasks were considered, including frequently occurring and safety critical tasks, exceptional or emergency responses, tasks where novice users may make mistakes or where errors are known to be common, and maintenance/inspection and moving tasks. The task [9] requires a thorough understanding of the work, so when developing the Usability Testing Protocol, existing procedures and documentation from three different models of ventilators were used to generate hierarchical task analyses which were used by the clinicians to develop the task scenario (Table 1).

**Table 1:** Task scenario for usability testing

Tasks	Participants (N = Nurse, D = Doctor)	Detailed sub-tasks	Equipment/keys/knobs/dials/screen, etc.
Ventilator set up and check prior to receiving patient			
Assemble circuit	N1 + D1	Check for integrity of valves/diaphragms, etc.	Ventilator; test equipment (e.g. test lung, flow sensor calibration equipment); power supply
Install circuit onto ventilator		Connect to test simulator (test lung) and perform self-test	
Set up ventilator to patient-specific parameters		Choose mandatory mode, set inspiratory pressure or tidal volume (IBW based) according to mode. Respiratory rate, I:E ratio (if adjustable) FiO_2_ and PEEP	
Check alarms (disconnect, high pressure, apnoea, volume alarms, O_2_ supply and battery level). Change alarm parameters		Disconnect, high pressure, apnoea, volume alarms, O_2_ supply and battery level. Change alarm parameters.	
Perform leak test and test patency of circuit with all parts attached (incl. filters)			
Check integrity and function of flow sensors Oxygen calibration			
Initiation of mechanical ventilation and adjust to initial parameters			
Intubation of patient, attach to ventilator, initiating and confirming safe ventilation.	N1 + D1 + D2 + runner	Complex process, separate evaluation, outside of scope of this evaluation	Airway trolley; ventilator; monitor; Sim Man/lung
Initiate ventilation and confirm safe delivery of set ventilator parameters	N1 or D1	Assess tidal volume, peak/plateau airway pressure, PEEP, FiO_2_, respiratory rate as displayed by ventilator	
Adjust respiratory rate and I:E ratio (if adjustable)	N1 or D1		
Rapidly increase or decrease FiO_2_	N1		
Optimise PEEP	N1 or D1	Sequential adjustments to improve oxygenation and titrate to compliance	
React to sudden change in status and alarms			
Respond to low supply pressure alarm		Evaluate integrity of supply pressure, look for disconnection	
Respond to high airway pressure alarm		Systematic evaluation from patient to ventilator	Monitor; ventilator; Sim Man/lung
Respond to low airway pressure alarm (circuit or patient disconnection)		Systematic evaluation from patient to ventilator looking for leaks or disconnections	
Rapidly adjust FiO_2_ in response to desaturation or enable suction		Single button (O_2_ flush) or complex step involving adjustment of FiO_2_	
Respond to volume alarms		High Vt or low Vt or MV	
Respond to apnoea alarm		Ensure backup mode initiates	
Respond to low battery or power disconnection		Identify source of power	

Errors were identified from previous research [10] to use both as prompts during the task scenario walk/talk through and to develop the evaluation proforma (Table 2). Key error types identified included:

**Table 2:** Evaluation template (strongly agree (5), agree (4), neutral (3), disagree (2), strongly disagree (1))

Eneral appearance and transportation	5	4	3	2	1	NA
1. The ventilator system is too large and heavy to transport easily	□	□	□	□	□	□
2. The ventilator is very fragile and can be damaged during transportation	□	□	□	□	□	□
3. It is very easy to transport (handles, wheels, manoeuvrability etc.)	□	□	□	□	□	□
4. It is very easy to use the ventilator system during stretcher use	□	□	□	□	□	□
5. It is very easy to determine battery charge	□	□	□	□	□	□
6. It is very easy to set up the circuit	□	□	□	□	□	□
Starting up and adjusting the settings	5	4	3	2	1	NA
7. It is very easy to set the PSV with PEEP mode and apnoea ventilation	□	□	□	□	□	□
8. It is very easy to specify inspiratory flow (e.g. assist volume control)	□	□	□	□	□	□
9. It is very easy to identify inspiratory trigger sensitivity	□	□	□	□	□	□
10. It is very easy to set the volume modes	□	□	□	□	□	□
11. It is very easy to switch from PSV with PEEP in volume mode (CV or ACV)	□	□	□	□	□	□
12. The time taken to setup and programme the ventilator system was reasonable	□	□	□	□	□	□
Alarms	5	4	3	2	1	NA
13. It is very easy to identify pre-set alarm ranges	□	□	□	□	□	□
14. It is very easy to modify an alarm range	□	□	□	□	□	□
15. It is very easy to identify the alarm(s) e.g. audio, visual alarms	□	□	□	□	□	□
16. The automatic alarms are very useful	□	□	□	□	□	□
17. It is very easy to cancel/reduce alarm sound	□	□	□	□	□	□
18. The error messages are meaningful	□	□	□	□	□	□
Interface	5	4	3	2	1	NA
19. The overall interface (screen, knobs, dials) is very easy to use	□	□	□	□	□	□
20. It is very easy to read/interpret the display from a distance	□	□	□	□	□	□
21. The plots are very useful	□	□	□	□	□	□
22. It is very easy to identify patient parameters	□	□	□	□	□	□
23. I think that I would need the support of a technical person to be able to use this system	□	□	□	□	□	□
24. I found the various functions in this system were well integrated	□	□	□	□	□	□
25. There are an acceptable number of menus to navigate to find what you need easily	□	□	□	□	□	□
Instructions for use and job aids	5	4	3	2	1	NA
26. The Instructions for use are very legible and clear	□	□	□	□	□	□
27. It is very easy to identify critical steps and required actions	□	□	□	□	□	□
28. It is very clear what I should do if the ventilator fails	□	□	□	□	□	□
29. I would imagine that most people would learn to use this system very quickly	□	□	□	□	□	□
30. It is very easy to learn how to use the ventilator system without a manual (instructions for use)	□	□	□	□	□	□
Overall feedback	5	4	3	2	1	NA
31. I thought the system was very easy to use	□	□	□	□	□	□
32. I think that I would like to use this system frequently	□	□	□	□	□	□
33. I found the system unnecessarily complex	□	□	□	□	□	□
34. I thought there was too much inconsistency in this system	□	□	□	□	□	□
35. I felt very confident using the system	□	□	□	□	□	□
36. I will need to learn a lot of things before I could get going with this system	□	□	□	□	□	□
37. The number of steps required to programme the ventilator system was acceptable	□	□	□	□	□	□
38. This ventilator system will be very safe to use on a patient	□	□	□	□	□	□

- **Failure to set up correctly**: including ability to use, despite failure to pass self-test; ability of novice to set up ventilator circuit according to on-screen instructions; inter-changeability of circuit with other types of ventilator circuitry that look similar.
- **Failure to find a setting site or display site**: difficulty with indirect adjustment of a requested setting; difficulty manipulating multiple controls of different types; difficulty making basic adjustments; confusion and error for the new or occasional user when adjusting for advanced parameters.
- **Setting site identified correctly but inappropriate setting**: illogical default settings, not necessarily immediately obvious to user; errors in adjusting the inspiratory trigger; unclear indication on the controls of the trigger sensitivity where changing one parameter leads to change in other parameters which is not immediately recognised.
- **Failure to confirm settings**: poor tactile and visual interface design/feedback.
- **Errors of interpretation**: difficulty in reading/interpreting display linked to information design and mode presentation (thresholds, configuration, default values, etc.).
- **Errors of cleaning**: risks associated with poor cleaning or failure to replace contaminated parts, missing parts during reassembly.
- **Errors of maintenance**: lack of knowledge (i.e. training/qualifications) of technical support staff; lack of awareness of common failures and failure modes.

## Formative usability testing

As this was a rapid manufacturing project, the Government specification only allowed for one day of formative usability testing [5]. To support the manufacturers, the CIEHF produced a task scenario (Table 1) and patient profiles to provide end users with the opportunity either to undertake simulated tasks with the physical prototype (walk-through) or to talk-through for an online evaluation. The development of the usability protocol included telephone assistance by CIEHF expert group members with the RMVS manufacturing teams.

The task scenario and patient profiles used previously published templates [11] and were developed by the clinicians on the CIEHF writing team (AW, A-MB and PM). The task scenario was designed as a pathway to reflect an individual patient requirement for ventilator use. It depicts a combined set of patient pathways to test the ventilator across a range of circumstances that would be unlikely to occur in an individual patient experience. The scenario starts from admission and initial testing of the ventilator, initiation of mechanical ventilation, mandatory modes (likely to be used in the initial phase), switching to spontaneous/triggered modes, monitoring and then finishes with the weaning process.

Patient profiles were developed to reflect common issues and patient presentations. Each profile included details about the patient, the task and the equipment to be used, for example:

- **Patient**: 62-year-old male, COVID positive, assessed by ICU consultant as deteriorating and tiring. Decision has been made to transfer to ICU for intubation and ventilation and ICU care. Standard operating procedure (SOP) requires transfer in full PPE and intubation and stabilisation in a dedicated area on ICU before transfer to bed space.
- **Task**: Set up ventilator, intubate patient and re-programme ventilator based on feedback once patient ventilated (e.g. changing respiratory rate, tidal volumes, positive end expiratory pressure (PEEP) according to values on ventilator, ETCO_2_ trace, oxygen saturations and arterial blood gases).
- **Equipment to be used**: patient bed, transfer monitor, ventilator under test and tubing, arterial and central venous pressure (CVP) transducer sets, intubation equipment including face mask, airway adjuncts, video laryngoscope, bougie, range of endotracheal tube (ETT) sizes, ETCO_2_ monitoring, tube ties, heat and moisture exchanger (HME) filter, waters’ circuit, airway rescue trolley, Naosgastric (NG) tube, drip stand, full PPE for aerosol generating procedures, intubation drugs.

## User evaluation questionnaire

A user evaluation questionnaire was developed based on previous research [10, 12–14] to provide a standardised template for gathering the required formative feedback from end users. The questions were checked against the task scenario and professional practice by the clinical authors and adapted to align with the MHRA Specification [5].

Finally, an issue reporting template was designed to support the systematic collection and recording of issues, including:

- Issue ID
- What was being tested (task)
- Task step or system function
- Issue description (and additional information, photo, video clip, etc.)
- Issue severity (used for prioritisation) should be agreed with the multidisciplinary design team before testing. A fatality, for example, would be classed as high severity
- Recommendation (proposed solution)
- Action or closure status (open/closed/rejected)

## Conclusion

This was a global crisis; everyone was trying to help and to adapt. As new players entered the field (i.e. manufacturers with engineering knowledge but unfamiliar with healthcare), it was important that efforts to respond to the crisis were based on established practice and structured approach [15]. Clinical staff working in ICUs and at the new National Health Service field hospitals could have been asked to use different types of ventilators with known risks of accidently pressing the wrong buttons or misreading information on screens.

The CIEHF community responded by providing structured guidance to help manufacturers with the novel requirements and challenges. The CIEHF guidance was issued to RMVS manufacturers to support the design and testing of new machines and to encourage standard designs and protocols to prevent avoidable harm to patients. The usability testing protocol supported realistic testing (work-as-done), including operability whilst wearing a range of PPE. The guidance and usability evaluation protocol are simple tools with the potential to make a significant contribution and could be adapted to other medical devices or equipment. This opens debate for national policymakers and others about the role and contribution of HFE in healthcare, which should be sustained beyond the immediate COVID-19 pandemic.

## References

[1] Department for Business, Energy & Industrial Strategy, UK *Call for Businesses to Help Make NHS Ventilators*. https://www.gov.uk/government/news/production-and-supply-of-ventilators-and-ventilator-components (18 May 2020, date last accessed).

[2] NHS, UK *Design for Patient Safety*. https://webarchive.nationalarchives.gov.uk/20171030124501/http://www.nrls.npsa.nhs.uk/resources/collections/design-for-patient-safety/ (18 May 2020, date last accessed).

[3] IEC. ISO 62366-1 *Medical Devices – Part 1: Application of Usability Engineering to Medical Devices*. Geneva: International Organization for Standardization, 2007.

[4] MHRA, UK *Human Factors and Usability Engineering – Guidance for Medical Devices Including Drug-Device Combination Products: V1* https://assets.publishing.service.gov.uk/government/uploads/system/uploads/attachment_data/file/645862/HumanFactors_Medical-Devices_v1.0.pdf (10 March 2020, date last accessed).

[5] MHRA, UK *Specification for Ventilators to be Used in UK Hospitals during the Coronavirus (COVID-19) Outbreak*. https://www.gov.uk/government/publications/specification-for-ventilators-to-be-used-in-uk-hospitals-during-the-coronavirus-covid-19-outbreak (10 March 2020, date last accessed).

[6] Chartered Institute of Ergonomics & Human Factors, UK *Human Factors in the Design and Operation of Ventilators for COVID-19*. https://bit.ly/HFandVentilators (22 April 2020, date last accessed)

[7] Chartered Institute of Ergonomics & Human Factors, UK *Formative Usability Testing for Rapidly Manufactured Ventilator Systems by Chartered Ergonomist and Human Factors Specialists (C.ErgHF)* https://bit.ly/VentilatorUsabilityV2 (22 April 2020, date last accessed).

[8] Phansalkar S , Edworthy J, Hellier E et al. A review of human factors principles for the design and implementation of medication safety alerts in clinical information systems. *J Am Med Inform Assoc* 2010; 17: 493–501. 20819851 10.1136/jamia.2010.005264 PMC2995688

[9] Stanton NA Hierarchical task analysis: developments, applications, and extensions. *Appl Ergon* 2006; 37: 55–79. 16139236 10.1016/j.apergo.2005.06.003

[10] Templier F , Miroux P, Dolveck F et al. Evaluation of the ventilator-user interface of 2 new advanced compact transport ventilators. *Respir Care* 2007; 52: 1701–9. 18028560

[11] Hignett S , Lu J, Fray M Two case studies using mock-ups for space planning in adult and neonatal critical care facilities. *J Healthc Eng* 2010; 1: 399–414.

[12] Jiang M , Liu S, Gao J et al. Comprehensive evaluation of user interface for ventilators based on respiratory therapists’ performance, workload, and user experience. *Med Sci Monit* 2018; 24: 9090–101. 30552313 10.12659/MSM.911853 PMC6319161

[13] Marjanovic N , L’Her E A comprehensive approach for the ergonomic evaluation of 13 emergency and transport ventilators. *Respir Care* 2016; 61: 632–9. 27121622 10.4187/respcare.04292

[14] Morita PP , Weinstein PB, Flewwelling CJ et al. The usability of ventilators: a comparative evaluation of use safety and user experience. *Critical Care* 2016; 20: 263. 10.1186/s13054-016-1431-1 PMC4992292 27542352

[15] Lintern S *Coronavirus: Lives Could be at Risk from Thousands of New Ventilators for the NHS, Warn Safety Experts*. https://www.independent.co.uk/news/health/coronavirus-uk-nhs-patient-safety-ventilators-intensive-care-a9457176.html (15 April 2020, date last accessed).

